# Preparation of Magnetic Carbon Composite from Waste Amine-Oxime Resin and Its Adsorption Properties for Chromium

**DOI:** 10.3390/ma18133066

**Published:** 2025-06-27

**Authors:** Haoyu Wang, Xianzhuo Su, Hongdan Yu, Yuhang Yuan, Jing Wu, Wenchao Yang, Chunlin He

**Affiliations:** 1 Guangxi Key Laboratory of Processing for Non-Ferrous Metals and Featured Materials, Key Laboratory of High Performance Structural Materials and Thermo-Surface Processing, School of Resources, Environment and Materials, Guangxi University, Nanning 530004, China; 2 Technical Innovation Center of Mine Geological Environmental Restoration Engineering in Southern Karst Area, Ministry of Natural Resources, Nanning 530028, China; 3 Natural Resources Ecological Restoration Center of Guangxi Zhuang Autonomous Region, Nanning 530029, China

**Keywords:** amidoxime chelating resin, carbonization, magnetism, adsorption, chromium

## Abstract

A waste amidoxime chelate resin (WAR) was converted into a magnetic composite adsorbent (MCA) via carbonization and magnetization for the effective removal of Cr(VI). Under optimized conditions (pH = 1, 30 °C, 1 h), the adsorbent achieved a maximum Cr(VI) adsorption capacity of 197.63 mg/g. The adsorption process conformed to the pseudo-second-order kinetic model (R^2^ > 0.98) and Langmuir isotherm model (R^2^ > 0.99). The materials can be separated by magnetism. The primary mechanism for the adsorption of Cr(VI) involved monolayer chemisorption. FTIR spectroscopy confirmed the dominant role of -C=O, C-O, and Fe-O in the adsorption process. XPS spectroscopy confirmed the dominant role of -C=O and C-O in the adsorption process. The successful conversion of the WAR into an MCA not only mitigates waste accumulation but also provides a cost-effective strategy for heavy metal remediation.

## 1. Introduction

Amidoxime chelating resin is a type of polymer with polyacrylonitrile, polyvinyl chloride, and polystyrene as the carbon-based skeleton. It is connected by a crosslinking agent so that the amino (−NH_2_) and oxime group (=N−OH) are attached to the same carbon atom [1,2]. Amidoxime resin is an amphoteric polymer compound. Under acidic conditions it can be complexed with anions [3]. In alkaline solutions, the high concentration of OH^−^ promotes deprotonation of the hydroxyl group in the amidoxime resin to form =N-O^−^. This anionic group can chelate metal cations by coordinating with their empty orbitals to form stable complexes. Due to its high selectivity, the resin effectively adsorbs target metal ions even in multi-ion coexisting systems, enabling the efficient separation of specific elements [4]. Due to its stable chemical properties, strong acid and alkali resistance, and large saturated adsorption capacity, amidoxime resin is widely used in the separation and purification of dispersed metals, the recovery and treatment of radioactive elements [5], and the treatment of wastewater and the recovery of heavy metal ions in water [6,7,8,9,10]. For example, amidoxime chelating resin is utilised for gallium adsorption from Bayer’s solution. It is discarded after being reused 50–60 times a month; 225 tons of waste resin is produced and shelved each year [11,12]. The traditional disposal methods of waste resin includes, but is not limited to, incineration and landfill [13,14]. However, landfilling consumes significant land resources, while incineration releases harmful pollutants. These contradict the green environmental policies and carbon neutrality goals adopted by many nations [15]. Recently, the pyrolysis of waste resins to synthesize diverse carbon-based materials has emerged as a prominent research focus [16,17,18,19,20]. Numerous studies have utilized waste ion-exchange resins as precursors to fabricate functional materials for gas storage, CO_2_ capture, and adsorption applications. Alternatively, other approaches involve reagent-mediated regeneration of waste resins while preserving their intrinsic organic framework.

In this study, waste amidoxime chelate resin (WAR) was converted into a magnetic composite adsorbent (MCA) via carbonization and magnetization for the effective removal of Cr(VI). We propose an innovative approach to utilize discarded resins for the preparation of magnetic adsorption materials, aligning with the principle of “treating waste with waste.” By repurposing waste resin materials, which would otherwise contribute to environmental pollution, we synthesized cost-effective and environmentally friendly magnetic adsorbents for the efficient adsorption, separation, and recovery of chromium (Cr) from industrial wastewater, particularly in electroplating effluents.

## 2. Materials and Methods

### 2.1. Reagents, Materials, and Apparatus

The waste amidoxime resin (WAR) was obtained from the Guangxi branch of Chalco (Pingguo, Baise, Guangxi, China). It had a particle diameter of 0.2−0.5 mm and a moisture content of 54.7%. The waste resin was thoroughly washed with deionized water three times, followed by drying in an oven at 50 °C for 24 h to prepare the raw material for adsorbent synthesis.

The experimental setup employed the following glassware and equipment: beakers (50, 100, 500 mL), test tubes (10, 50 mL), volumetric flasks (50, 100, 1000 mL), syringes (5 mL), pipettes (1, 5, 10 mL), ceramic crucibles (10, 50 mL), glass vials (30, 50 mL), graduated cylinders (10, 50 mL), Petri dishes, glass rods, and mortar and pestle sets. The chemical reagents used in this study are listed in Table 1. The apparatus used in the experiment is shown in Table 2.

### 2.2. Preparation of Materials

#### 2.2.1. Carbonization

The WAR underwent sequential pretreatment steps. First, it was washed three times with deionized water and dried at 50 °C for 24 h. For chemical activation, 5 g of the WAR was mixed with 50 mL of 40% H_3_PO_4_ in a 100 mL beaker and stirred at 500 rpm in a 40 °C water bath for 4 h. The H_3_PO_4_-treated resin was then washed with deionized water, dried at 50 °C for 24 h, subsequently impregnated with ZnCl_2_ at a 1:1 mass ratio, and then cured in an oven at 80 °C for 12 h, from which we obtained the modified material (MDM). Adding ZnCl_2_ during the carbonization process can effectively increase the specific surface area, and also increase the presence of oxygen-containing functional groups (such as carboxyl groups and phenolic hydroxyl groups), thereby enhancing the adsorption capacity of the MCA for Cr(VI). Subsequently, the MDM was treated by pyrolysis and carbonization.

The pyrolysis was conducted in a tube furnace under a N_2_ atmosphere (0.1 L/min flow rate), where the sample was heated to 500 °C at 3 °C/min and held for 2 h before natural cooling. The carbonized material was then treated with 0.1 M HCl, washed to neutral pH, and finally dried at 80 °C for 24 h to obtain the activated carbon product (ACP).

#### 2.2.2. Preparation of the MCA

**Figure 1:**
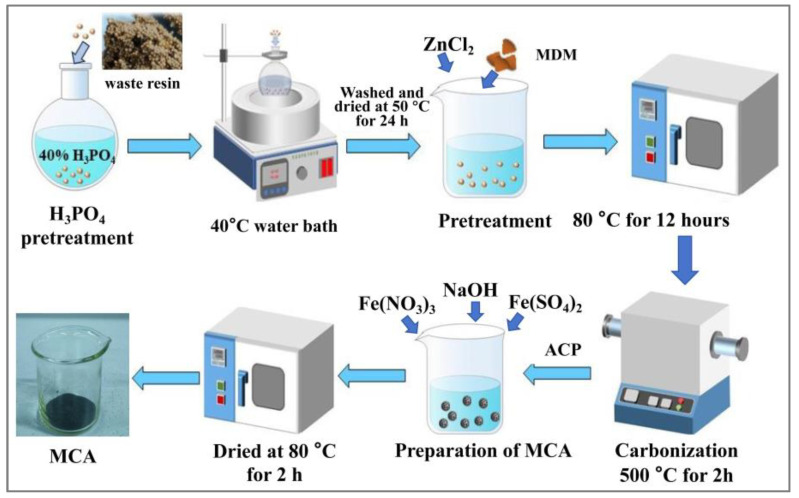
Preparation flowchart of the MCA.

Synthesis of the magnetic composite material (MCA) involved the following steps: 10 g of the ACP was added into a beaker containing 100 mL of 0.6 mol/L Fe(NO_3_)_3_ and 0.30 mol/L Fe(SO_4_)_2_ solution. The pH was adjusted to 7 using NaOH, and the suspension was sonicated for 2 h to ensure homogeneity. The mixture was allowed to stand at room temperature (protected from light) for 24 h. Finally, it was dried at 80 °C for 24 h. The dried sample was ground into a fine powder using a mortar and pestle to obtain the MCA. The whole preparation flowchart is shown in Figure 1.

### 2.3. Experimental

#### 2.3.1. Adsorption Conditions and Experimental Parameters

A single-variable method was employed to evaluate the adsorption performance of the magnetic activated carbon for Cr(VI) under varying conditions. Solution preparation used the following steps: a 300 mg/L Cr(VI) stock solution was prepared in a volumetric flask. The solution pH was adjusted using 1 mol/L HCl or 1 mol/L NaOH. Precisely 0.05 g of the MCA was weighed and combined with 20 mL of the Cr(VI) solution in a 30 mL glass bottle. The mixture was secured with rubber bands and agitated in a constant temperature shaker at 140 rpm for optimal contact. The suspension was then filtered through a 0.45 μm membrane attached to a 5 mL syringe to separate the adsorbent. The Cr(VI) concentrations before (*C*_1_) and after (*C*_2_) adsorption were measured by an inductively coupled plasma-atomic emission spectrometry (ICP-AES) (ICPS-750, SHIMADZU, Kyoto, Japan). The adsorption capacity (Q, mg/g) and removal efficiency (E, %) were calculated as follows:

(1) $$Q=\frac{(C_{1}-C_{2})\times V}{m}$$

(2) $$E=\frac{\left(C_{1}-C_{2}\right)}{C_{1}}\times 100\%$$

where *V* (L) is the total volume of the Cr(VI) mother liquor used, and *m* (g) is the mass of the adsorbent used.

#### 2.3.2. The Model Fitted to the Data Used in the Experiment

In this experiment, quasi-first-order (PFO), quasi-second-order (PSO), and Weierstrass-Mandelbrot (W-M) functions were used to fit the experimental data obtained from the adsorption data [21,22,23,24]. The equations are given below:

(3) $$\ln(Q_{e}-Q_{t})=\ln Q_{e}-k_{1}t$$

(4) $$\frac{t}{Q_{t}}=\frac{1}{k_{2}Q_{e}^{2}}+\frac{t}{Q_{e}}$$

(5) $$Q_{t}=k_{d}t^{0.5}+B$$

where *Q_e_* (mg/g) is the adsorption capacity of the absorbent for Cr(VI), *Q_t_* (mg/g) is the adsorption capacity at time *t* (min), *k*_1_ (min^−1^) is the quasi-first-order kinetic rate constant, *k*_2_ (g/mg/min) is the quasi-second-order kinetic rate constant, and *k*_d_ (mg/g/min^0.^^5^) is the intraparticle diffusion rate constant.

The four adsorption isotherm models, Langmuir [25], Freundlich [26], Temkin [27], and Dubinin-Radushkevich (D-R model) [27,28,29] were used in this experiment to investigate the adsorption behavior of the MCA adsorbent on Cr(VI). The equations are given below.

(6) $$\mathrm{Liner}\;\mathrm{Langmuir}\;\mathrm{model}:\;\frac{C_{e}}{Q_{e}}=\frac{C_{e}}{Q_{m}}+\frac{1}{K_{L}Q_{m}}$$

(7) $$\mathrm{Liner}\;\mathrm{Freundlich}\;\mathrm{model}:\;\ln Q_{e}=\ln K_{f}+\frac{\ln C_{e}}{n}$$

(8) $$\mathrm{Temkin}\;\mathrm{model}:\;Q_{e}=\frac{RT}{K_{t}}\ln(AC_{e})\;\begin{array}{c} \mathrm{Temkin}\;\mathrm{model}:\;Q_{e}=B\ln A+B\ln C_{e}\\ B=\frac{RT}{K_{t}} \end{array}$$

(9) $$D-R\;\mathrm{model}:\;Q_{e}=Q_{m}\exp(-\beta \varepsilon^{2})$$

(10) $$\varepsilon =RT\ln(1+\frac{1}{C_{e}})$$

where *Q_m_* (mg/g) is the saturated adsorption capacity; *C_e_* (mg/L) is the equilibrium Cr(VI) concentration; *K_L_* (L/mg) is the Langmuir constant, *K_f_* (mg^1−n^·L^n^·g^−1^) is the Freundlich constant; *n* is the intensity associated with adsorption. Both *K_t_* and *A* are constants; *Q_m_* (mg/g) is the saturated adsorption capacity in the D-R model; *β* (mol^2^/J^2^) is the constant related to the adsorption energy; and *ε* (J/mol) is the Polanyi potential.

Adsorption thermodynamics were used to study the change in adsorption energy of an adsorbent during adsorption, as shown in the following equation:

(11) $$\ln K_{s}=\frac{\Delta S}{R_{0}}-\frac{\Delta H}{R_{0}T}$$

(12) ΔG=ΔH−ΔST

where Δ*S* (J/(mol·K)), Δ*H* (kJ/mol), and Δ*G* (kJ/mol) are the entropy change, enthalpy change, and Gibbs free energy, respectively; *K*s is the distribution coefficient; *T* (K) is the Kelvin temperature; and *R*_0_ is the universal gas constant with the value of 8.314 J/(mol·K).

#### 2.3.3. Material Characterization and Mechanism Analysis Methods

To characterize the surface morphology and elemental content of the adsorbent before and after adsorption, a scanning electron microscope energy spectrometry all-in-one machine (SEM-EDS, Phenom Pro 800-07334, Eindhoven, The Netherlands) with a high-brightness CeB_6_ filament was used.

To characterize the changes in functional groups of the adsorbent before and after adsorption, the adsorbent in the paper was analyzed by infrared spectroscopy (FTIR, IRTracer-100, Shimadzu Corporation, Kyoto, Japan). Infrared spectroscopy determines molecular composition and functional group structure [30]. Before the test, the sample was ground into a powder and fully dried in an oven at 50 °C, and then fully ground and mixed with KBr powder in the ratio of 1:100 and then pressed to test.

A simultaneous thermal analyzer (STA449F 3, NETZSCH, Selb, Germany) was used to characterize the thermal decomposition behavior of the materials, and to analyze the mass loss and energy changes of the adsorbent at different temperatures. This investigated the thermal stability and decomposition process of the materials. The test conditions were as follows: temperature increase rate of 10 °C/min, temperature interval of 25–800 °C, and pyrolysis under a N_2_ atmosphere.

Brunauer-Emmett-Teller measurements (BET, Micromeritics, Norcross, USA) were used to characterize the specific surface area and pore size distribution of the adsorbent. The conditions were as follows: a small amount of adsorbent powder was weighed and degassed with N_2_ at 80 °C for 12 h to remove the water retained on the surface of the adsorbent and in the pores, and the N_2_ sorption-desorption experiments were carried out at a critical temperature of 77K [31].

The elemental composition and surface morphology of the sample were analyzed using a thermoelectric XPS (XPS, ESCALAB 250Xi, Waltham, MA, USA). The magnetic properties of the adsorbent were characterized by measuring its hysteresis loop using VSM (Vibrating Sample Magnetometer, Quantum Design, San Diego, CA, USA).

## 3. Results and Discussion

### 3.1. SEM-EDS

**Figure 2:**
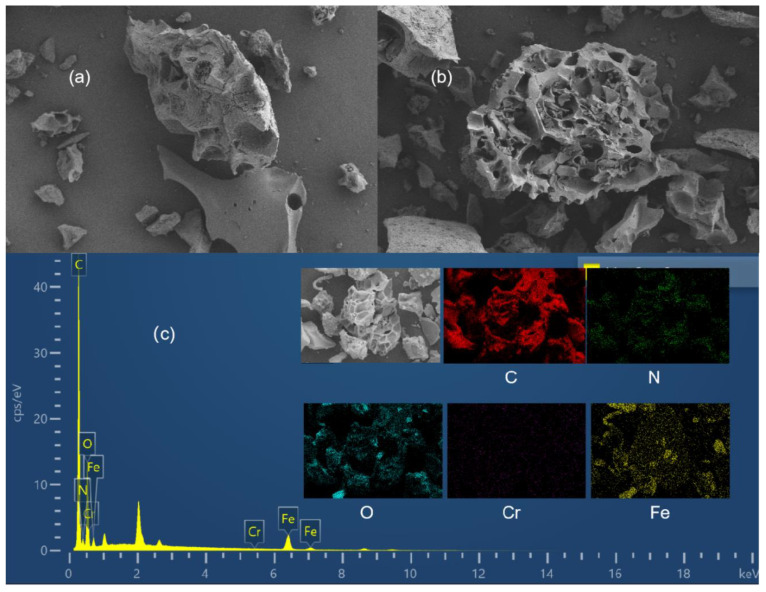
SEM and EDS analysis of MCA after Cr(VI) adsorption: (**a**,**b**) SEM images of MCA, (**c**) EDS spectrum post Cr(VI) adsorption.

The surface morphology of the MCA was observed by SEM and the elemental content of the adsorbent was probed by EDS, as shown in Figure 2. It can be seen that the MCA presents as an irregular block structure, which is made up of many small irregularly-shaped particles stacked together. During the process of stacking, the material forms an irregular pore structure, which increases the surface area of the material and provides more adsorption sites for the adsorption of ions.

Table 3 shows that the MCA is mainly composed of the elements C, N, O, and Fe. At the same time, the adsorbent detected the element Cr(VI) after adsorption, which indicates that Cr(VI) was successfully adsorbed by the MCA.

### 3.2. TG-DSC and Magnetism

**Figure 3:**
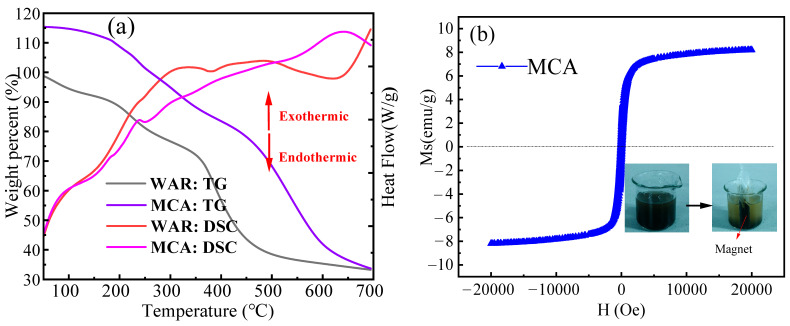
(**a**) Thermogravimetric diagrams of three adsorbents and DSC analysis of three adsorbents; (**b**) Magnetic hysteresis loop of the MCA.

A thermogravimetric differential thermal analysis of MAC was carried out by applying a differential thermal-thermogravimetric simultaneous analyzer. The samples were heated up to 800 °C at a ramp rate of 5 °C/min. The thermogravimetric differential thermal analysis was performed on the untreated waste resin (WAR) and MAC, as shown in Figure 3a. The thermal degradation process occurs in three distinct stages, as follows. Stage I (50–200 °C): a mass loss of approximately 9.3% occurs, accompanied by an endothermic peak in the DSC curve. This stage is attributed to the volatilization of residual water molecules trapped within the resin’s internal cavities and pores. Stage II (200–350 °C): upon reaching 200 °C, the pyrolysis enters its second stage. A distinct exothermic peak emerges on the DSC curve, concurrent with significant mass loss. This stage corresponds to the decomposition of the resin’s polymeric backbone structure and its functional groups. Stage III (>350 °C): as the temperature further increases, a significant mass loss of approximately 30% occurs. This stage is associated with the breakage of styrene chains within the resin and cleavage of the C-H bonds on the benzene rings [32,33], leading to the formation of an amorphous carbon structure [34].

Treatment with H_3_PO_4_ and ZnCl_2_ significantly shifted the onset of Stage III pyrolysis from 350 °C (unmodified resin, WAR) to 550 °C. The pyrolysis process transitioned from the low-temperature region to the high-temperature region. Consequently, both Stage II and Stage III occurred over wider temperature ranges in the modified resin compared with the unmodified resin (WAR). The unmodified resin (WAR) exhibited a relatively concentrated exothermic process, releasing heat more rapidly. In contrast, the modified resin (H_3_PO_4_ and ZnCl_2_-treated) showed a broader exothermic peak, with the peak maximum shifting approximately 50 °C higher, and a smoother DSC curve profile. This shift to higher temperatures and a smoother exothermic profile indicates that the modified resin undergoes slower and more complete decomposition during heating. These controlled decomposition kinetics are beneficial for the development of a porous carbon structure.

As can be seen in Figure 3b, the hysteresis loop in the figure shows a very linear relationship [35], which usually means that the magnetic response of the material is nearly proportional to the external magnetic field. This phenomenon is more common in paramagnetic or antimagnetic materials, which show that the MCA is weakly magnetic. This means it can be magnetically separated and accumulated on one side of the magnet.

### 3.3. BET

**Figure 4:**
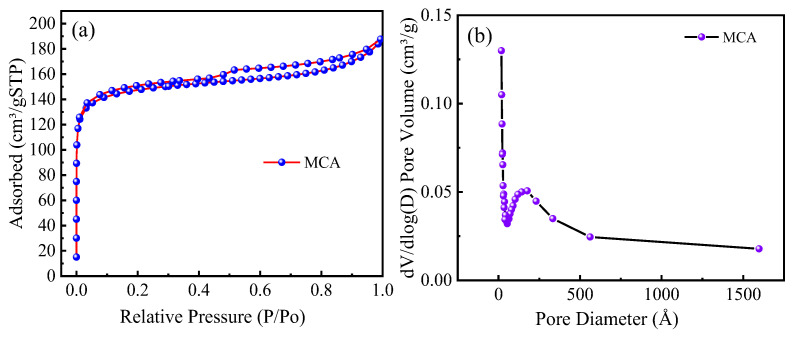
(**a**) N_2_ sorption-desorption isotherm of the adsorbents, (**b**) Pore size distribution of the adsorbents.

Figure 4a shows the N_2_ sorption-desorption isotherms and hysteresis loop curves of the MCA at relative pressures. It can be seen that the MCA conforms to the type II isotherm model and the H_4_ hysteresis loop model, respectively. This indicates that these two adsorbents have a non-porous structure, and the adsorption phenomenon occurs due to the interactions between the surface of the adsorbent and the adsorbate. It can be seen from Figure 4b that the distribution of the pore structure of the MCA is more concentrated, and the pore size of the MCA is mainly concentrated around 20 nm. The specific surface area and pore size are shown in Table 4. The specific surface area of the MCA is 569.89 m^2^/g, indicating that the MCA has a large specific surface area, with an average pore size of 20.38 nm.

### 3.4. Adsorption

#### 3.4.1. Effect of Initial pH and Temperature on Adsorption

**Figure 5:**
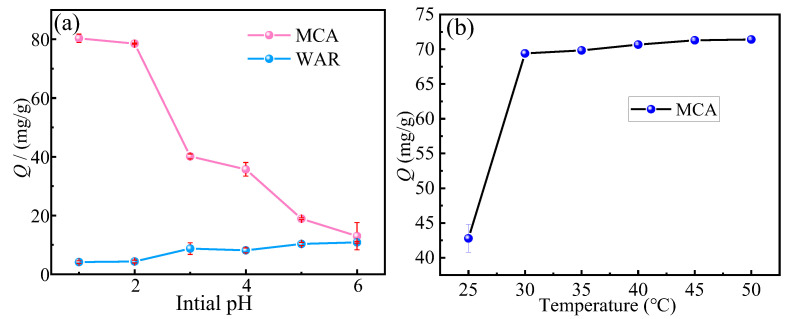
(**a**) Effect of initial pH on adsorption capacity, (**b**) effect of temperature on adsorption capacity.

Solution pH is a primary factor influencing adsorption performance because it governs both the surface charge of the MAC adsorbent and the availability of metal-binding sites. As depicted in Figure 5a, the Cr(VI) adsorption capacity of the MAC decreases progressively with an increasing pH. Consequently, the optimum adsorption pH for the MAC is 1. This occurs because at higher pH values, elevated OH^−^ concentrations induce competitive adsorption between OH^−^ and Cr_2_O_7_^2−^ for the binding sites, thereby reducing chromium uptake.

Temperature significantly influences adsorption performance, primarily by affecting reaction kinetics and thermodynamic equilibrium. As shown in Figure 5b, the Cr(VI) adsorption capacity of the MAC increases with rising temperature until reaching a plateau above 50 °C. Consequently, 30 °C was identified as the optimum adsorption temperature under these experimental conditions. This plateau occurs because the available adsorption sites become saturated with chromium ions, preventing further capacity increases despite additional thermal energy.

#### 3.4.2. Adsorption Kinetic

**Figure 6:**
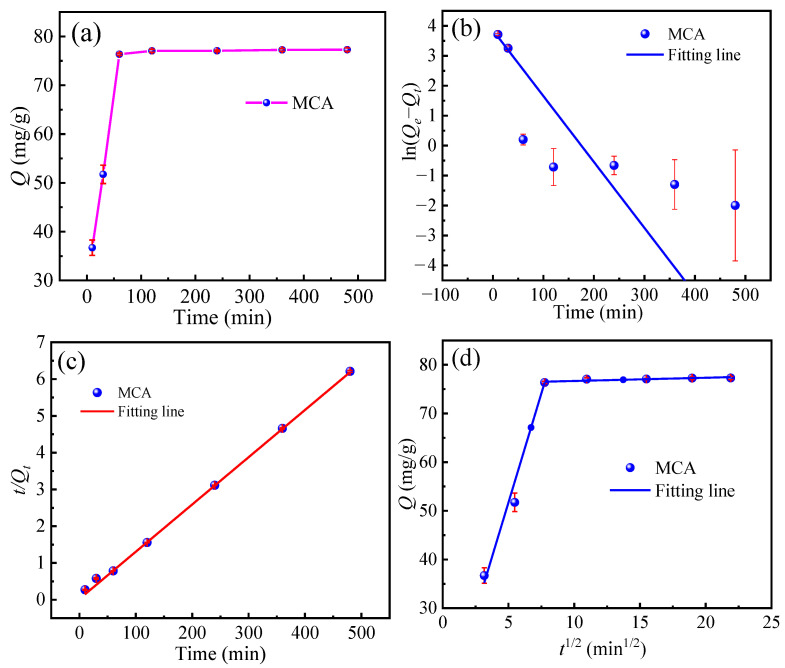
(**a**) Effect of adsorption time on adsorption capacity; (**b**) Fitting of the Pseudo primary model, (**c**) Fitting of the Pseudo secondary model; (**d**) Weber−Morris intraparticle diffusion model.

Adsorption kinetics is an important aspect in the evaluation of the adsorption performance of the MCA; therefore, the reaction kinetics at different temperatures were investigated (Figure 6a–d). As shown in Figure 6a, within the first 30 min there was a significant increase in adsorption capacity due to the accessible adsorption sites on the resin surfaces and the high concentration of Cr(VI). The MCA reached adsorption equilibrium after 1 h, achieving maximum Cr(VI) removal efficiency.

As shown in Figure 6b,c, the quasi-first-order kinetic model (PFO) and quasi-second-order kinetic model (PSO) were used to fit the adsorption data of the Cr(VI). It can be seen by comparing the fitted parameters in Table 5, that the correlation coefficient *R*^2^ of quasi-first-order fitting is only 0.68, and the quasi-second-order R^2^ of the kinetic model is 0.99. This indicates that the quasi second-order kinetic model is more consistent with the adsorption behavior of the adsorbent on Cr(VI), which suggests that the adsorption behavior of the MCA for Cr(VI) is mainly controlled by a chemical adsorption mechanism. As shown in Figure 6d, the adsorption kinetics of Cr(VI) onto the adsorbent were analyzed using the intraparticle diffusion model. The model revealed two distinct adsorption stages. Stage 1, driven by the high initial Cr(VI) concentration in solution and the abundant available active sites on the adsorbent, so the diffusion rate was highest resulting in the steepest slope and a rapid increase in adsorption capacity. Stage 2, as adsorption progressed, the Cr(VI) concentration decreased significantly and most of the active sites were occupied. This led to an increased diffusion resistance, a substantial decrease in the adsorption rate (evident as a much lower slope), and a gradual approach to adsorption equilibrium. Notably, the linear fits for both diffusion stages did not pass through the origin. This deviation indicates that intraparticle diffusion was not the sole rate-controlling step, and that boundary layer (film) diffusion also exerted a significant influence on the overall adsorption kinetics.

#### 3.4.3. Saturation Adsorption, Reuse Performance, and Adsorption Isotherm Modeling

**Figure 7:**
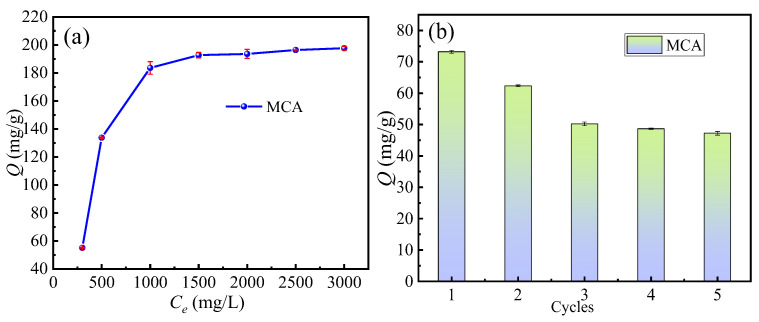
(**a**) Effect of initial concentration on adsorption capacity of the MCA; (**b**) Reuse performance.

Figure 7a depicts the effect of initial Cr(VI) concentration (ranging from 300 mg/L to 3000 mg/L) on the equilibrium adsorption capacity of the magnetic activated carbon (MCA) adsorbent. The adsorption capacity of the Cr(VI) by the MCA increased with a rising initial concentration, driven by the enhanced mass transfer driving force. Saturation of the adsorbent’s active sites was reached at an initial concentration of 1000 mg/L, evidenced by the plateau in the adsorption isotherm. Further increases in concentration beyond this point yielded no significant increase in adsorption capacity because the available sites were effectively saturated with chromium ions. The adsorbent achieved a maximum Cr(VI) adsorption capacity of 197.63 mg/g. To evaluate the adsorption capacity of the MCA, the adsorption capacity of the MCA was compared with other carbon-based adsorption materials, as shown in Table 6. It can be seen that the MCA has a significant advantage in adsorption capacity. This also indirectly demonstrates the superiority of the synergistic treatment process using H_3_PO_4_ and ZnCl_2_.

Figure 7b demonstrates the regeneration performance of the MCA over five consecutive adsorption-desorption cycles under identical conditions. The adsorption capacity gradually decreased with each cycle. Notably, the rate of decline diminished by the fourth cycle, and the capacity in the fifth cycle stabilized at a value of approximately 48 mg/g. This progressive confirms that the MCA retains significant regenerability and structural stability despite repeated use, and the Cr(VI) can be recovered. After deactivation from long-term use, it can continue to be activated and regenerated as a carbonaceous material for other adsorption applications.

**Figure 8:**
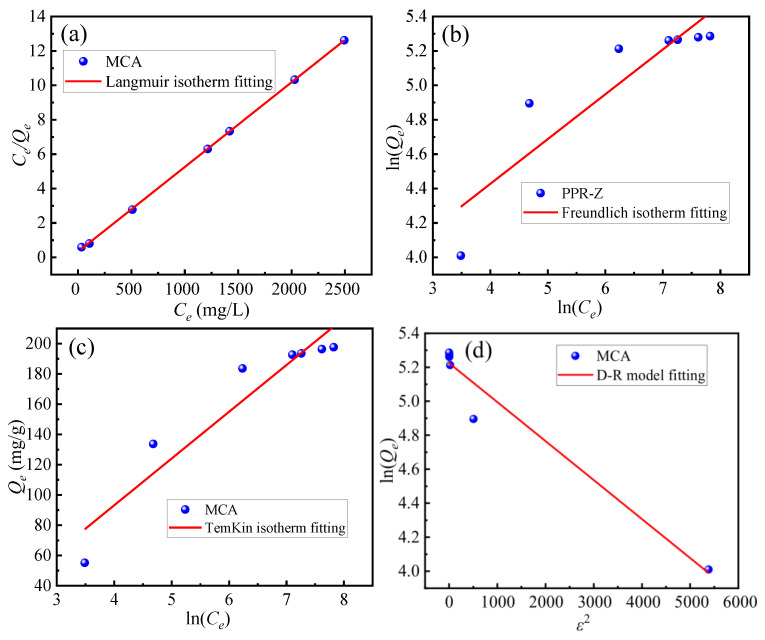
(**a**) Langmuir adsorption isotherm fitting, (**b**) Freundlich adsorption isotherm fitting, (**c**) TemKin adsorption isotherm fitting, (**d**) D-R model fitting.

Isotherm adsorption models are essential tools for evaluating adsorbent capacity and characterizing adsorbate-adsorbent interactions. Figure 8a–c presents the fitting of experimental adsorption data for Cr(VI) on the MCA to three isotherm models, including Langmuir, Freundlich, and Temkin. Analysis of the fitting parameters (Table 7) reveals a distinct model suitability. The Freundlich model exhibited moderate correlation (R^2^ = 0.82), while the Temkin model showed improved fitting (R^2^ = 0.91). Significantly, the Langmuir model demonstrated the highest correlation (R^2^ > 0.99), indicating it most accurately describes the adsorption behavior. This strong agreement with the Langmuir isotherm suggests Cr(VI) adsorption occurs primarily via monolayer coverage on homogeneous binding sites, with minimal interaction between adsorbed ions.

The adsorption energy was further investigated using the Dubinin-Radushkevich (D-R) model (Figure 8d). Excellent fitting (R^2^ =0.96 at 303 K) enabled determination of the mean free energy of adsorption (E). The calculated E value exceeded 8 kJ·mol^−1^, confirming the adsorption process is predominantly chemisorption. This indicates a chemical interaction between Cr(VI) ions and the functional groups on the MCA surface, consistent with the mechanism inferred from the kinetic modeling.

#### 3.4.4. Adsorption Thermodynamics

**Figure 9:**
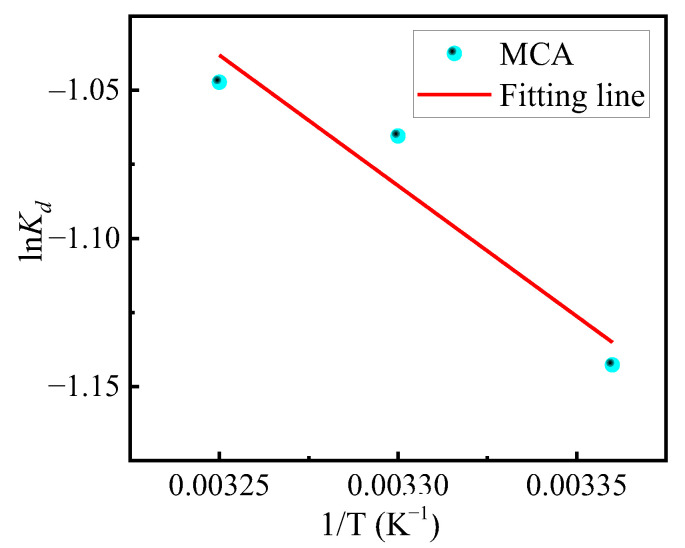
Relationship between lnK_d_ and 1/T.

The thermodynamics of the adsorption process is a key factor affecting the adsorption process and is also an important reference for understanding the spontaneity and energy changes of the adsorption process of adsorbents. Equations (11) and (12) were used to investigate the spontaneity and free energy changes of the adsorption behavior of the MCA during the adsorption of chromium ions in the presence of adsorption saturation, as shown in Figure 9.

The enthalpy (∆H) and entropy (∆S) of the adsorption process were calculated from the slope and intercept of the fitted plots to calculate the change of Gibbs free energy during adsorption, and to determine the spontaneity of the adsorption process and the change in the free energy, which is shown by the fitted parameters in Table 8. With an increase of the adsorption temperature from 298 K to 308 K, all of the Gibbs free energies were positive. Further, with the rise in temperature, the Gibbs free energy decreased from 2.802 kJ·mol^−1^ to 2.651 kJ·mol^−1^ during the adsorption process, indicating that the process of MCA adsorption of Cr (VI) is a non-spontaneous heat adsorption process. Thus, the rise in temperature makes the resistance to adsorption smaller, which is to some extent favorable to the adsorption process [46]. This explains the phenomenon that the rise in temperature makes the saturated adsorption capacity increase significantly from a thermodynamic point of view.

### 3.5. Adsorption Mechanism

**Figure 10:**
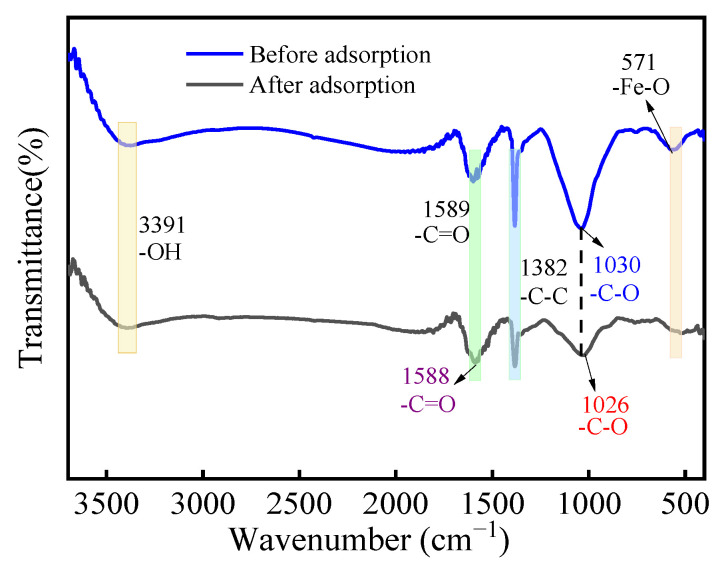
FTIR spectra of MCA before and after adsorption.

The FTIR spectra of the MCA before and after adsorption is shown in Figure 10. Before the adsorption, the absorption peaks for -OH, -C=O, -C-C, -C-O, and -Fe-O appeared at the positions of 3391 cm^−1^, 1589 cm^−1^, 1382 cm^−1^, 1030 cm^−1^, and 562 cm^−1^, respectively. Comparing the absorption peaks curves after the adsorption and desorption, after the adsorption, the absorption peaks of -Fe-O in the MCA were shifted from 562 cm^−1^ to 520 cm^−1^, indicating that -Fe-O interacted with the Cr(VI) during the adsorption process, resulting in the shift of the absorption peak. In summary, the FTIR analysis illustrated that the -Fe-O functional group played an important role in the adsorption process. FTIR spectroscopy confirmed the dominant role of -C=O, C-O, and Fe-O in the adsorption process.

**Figure 11:**
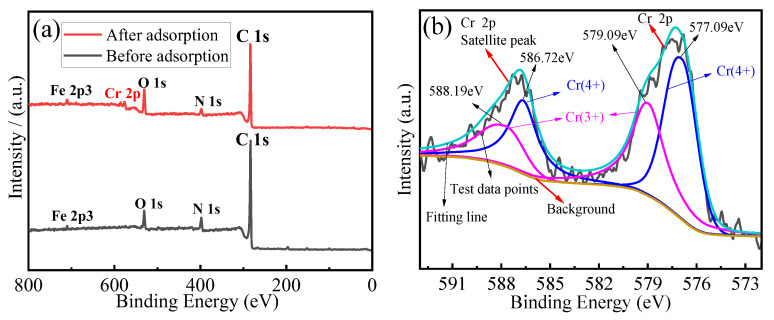

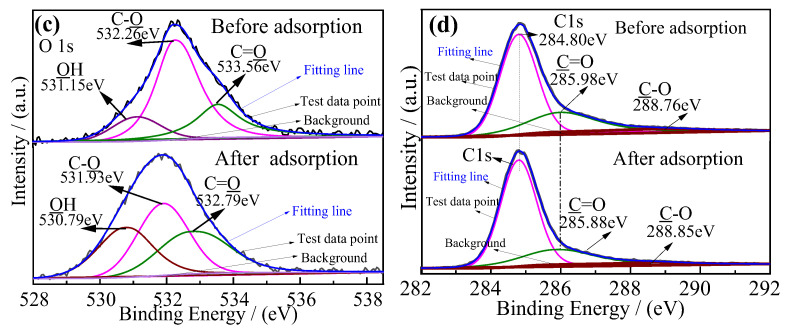
(**a**) Full spectrum of the MCA before and after adsorption, (**b**) Cr(VI) narrow spectrum of the MCA after adsorption, (**c**) Oxygen narrow spectrum of the MCA before and after adsorption. (**d**) Carbon narrow spectrum of the MCA before and after adsorption.

Figure 11a presents the XPS survey spectra of the MCA before and after Cr(VI) adsorption. Characteristic peaks corresponding to C 1s, N 1s, O 1s, Cr 2p and Fe 2p3 were observed at binding energies of 284 eV, 400 eV, 530 eV, 577 eV and 710 eV, respectively. After adsorption, a distinct Cr 2p peak appeared at 577 eV, confirming the successful adsorption of the Cr(VI) onto the MCA.

Analysis of the high resolution Cr 2p spectrum (Figure 11b) reveals the presence of the Cr in two valence states on the adsorbed MCA: Cr(III) (peaks at 579.09 eV and 588.19 eV) and Cr(VI) (peaks at 577.09 eV and 586.72 eV). The lower peak intensity for Cr(III) compared with Cr(VI), indicates that the adsorbed chromium primarily retains its hexavalent state. This suggests that under the prevailing conditions of low acid concentrations, the limited availability of H^+^ ions resulted in only a partial reduction of Cr(VI) to Cr(III) [11].

Figure 11c shows a significant shift in the binding energy of the carbonyl (-C=O) oxygen peak from 533.56 eV to 532.79 eV after Cr(VI) adsorption. In contrast, the O 1s peak associated with carboxyl/carbonyl carbon (-C-O/-C=O) in the high-resolution C 1s spectra (Figure 11d) exhibited a much smaller shift, changing only from 288.76 eV to 288.85 eV. The pronounced shift in the oxygen peak strongly suggests that the carbonyl oxygen atoms (-C=O) directly participate in chelation with Cr(VI) ions in solution. This interaction stabilizes the adsorbed complex, leading to a decreased binding energy (indicating a lower energy state) and the formation of a stable structure. FTIR and XPS spectroscopy confirmed the dominant role of -C=O and C-O in the adsorption process.

## 4. Conclusions

The adsorbent exhibits an irregular blocky morphology composed of aggregated smaller particles. Numerous slit-like pores are present on the surface and within the structure. The maximum Cr(VI) adsorption capacity of the MCA is 197.63 mg/g (at pH = 1, T = 303K, t = 1 h). The adsorption kinetics best fit a pseudo-second-order model. The adsorption isotherms followed the Langmuir model, suggesting monolayer adsorption dominated by chemisorption. Thermodynamics revealed the process is non-spontaneous and endothermic. An increasing temperature reduces the energy barrier, slightly favoring adsorption. The primary mechanism for the adsorption of the Cr(VI) involved monolayer chemisorption. FTIR spectroscopy confirmed the dominant role of -C=O, C-O, and Fe-O in the adsorption process. XPS spectroscopy confirmed the dominant role of -C=O and C-O in the adsorption process. The successful conversion of the WAR into an MCA not only mitigates waste accumulation but also provides a cost-effective strategy for heavy metal remediation.

## Figures and Tables

**Table 1:** Reagents used in the experiment.

Reagent Name	Molecular Formula	Reagent Grade	Company
Sodium hydroxide	NaOH	Analytical Reagent	Guangdong Guanghua Technology Co., Ltd., (Guangzhou, China)
Zinc chloride	ZnCl_2_	Analytical Reagent	Guangdong Guanghua Technology Co., Ltd., (Guangzhou, China)
Ferric nitrate	Fe(NO_3_)_3_	Analytical Reagent	Guangdong Guanghua Technology Co., Ltd., (Guangzhou, China)
Ethanol absolute	C_2_H_5_OH	Analytical Reagent	Guangdong Guanghua Technology Co., Ltd., (Guangzhou, China)
Potassium dichromate	K_2_Cr_2_O_7_	Analytical Reagent	Guangdong Guanghua Technology Co., Ltd., (Guangzhou, China)
Hydrochloric acid	HCl	Analytical Reagent	Sinopharm Chemical Reagent Co., Ltd., (Shanghai, China)
Phosphoric acid	H_3_PO_4_	Analytical Reagent	Sinopharm Chemical Reagent Co., Ltd., (Shanghai, China)
Nitric acid	HNO_3_	Analytical Reagent	Sinopharm Chemical Reagent Co., Ltd., (Shanghai, China)

**Table 2:** Apparatus used in the experiment.

Instrument	Instrument Model	Manufacturer
Fourier Transform Infrared Spectroscopy	IR Tracer 100	SHIMADZU (Kyoto, Japan)
SEM-EDS Phenom XL	Phenom Pro 800-07334	Phenom (Eindhoven, the Netherlands)
Differential Heat-Thermogravimetric Simultaneous Analyzer	STA449F 3	Netzsch (Selb, Germany)
Electronic Analytical Balance	AUY220	SHIMADZU (Kyoto, Japan)
Specific Surface Area Analyzer	TriStarII 3020	Micromeritics (Norcross, GA, USA)
Constant Temperature Drying Box	DG-410C	Chongqing Daho Technology Co., Ltd. (Chongqing, China)
Constant Temperature Water Bath Oscillator	NTS-4000BH	EYELA (Tokyo, Japan)
Thermostatic Water Bath	HCJ-4E	Changzhou Enpei Instrument Manufacturing Co., Ltd. (Changzhou, Jiangsu, China)
Pipette	Finnpipette	Thermo Fisher Scientific (Waltham, MA, USA)
Laboratory Water Purifier	Master-S15UV	Shanghai Hetai Instrument Co., Ltd. (Shanghai, China)
Tube Furnace	GXL-1700X	Hefei Kejing Instrument Co., Ltd. (Hefei, Anhui, China)
Ultrasonic Cleaner	XO-5200DTD	Nanjing Xianou Instrument Manufacturing Co., Ltd. (Nanjing, China)

**Table 3:** Element contents of the adsorbents before and after adsorption.

Element (%)	Magnetic Activated Carbon Adsorbent (MCA)	
	Original	Adsorption
C	71.19	71.06
N	8.15	8.36
O	19.39	18.91
Cr	0	1.43
Fe	1.26	0.23

**Table 4:** Surface area and pore size analysis.

Adsorbent	Langmuir (m^2^/g)	BET (m^2^/g)	Pore Volume (m^3^/g)	Pore Size (nm)
MCA	569.88	569.89	0.2904	20.38

**Table 5:** Kinetic model fitting parameters.

Adsorbent	PFO			PSO			W-M	
	*Q_e_*	*k*_1_	*R* ^2^	*Q_e_*	*k*_2_	*R* ^2^	*R*_1_^2^	*R*_2_^2^
MCA	77.6	0.022	0.68	77.6	0.58	0.99	0.99	0.81

**Table 6:** Amount of Cr(VI) adsorbed by different carbon-based adsorbents.

Adsorbents	Q/(mg/g)	Reference
MCA	197.63	This work
KSAC-CeO_2_	14.00	[36]
SLACM	227.70	[37]
AC-NH3-900	95.60	[38]
ACSs	230.15	[39]
chitosan	82.78	[40]
NTP	6.48	[41]
M-AC	58.39	[42]
ZVI@TBC	186.20	[43]
C-DE@S	142.83	[44]
MU-CNTs/Fe-700	23.70	[45]

**Table 7:** Isotherm adsorption line model fitting parameters of MCA.

Metal ion	Langmuir model			Freundlich model		
	*Q_m_*	*K_L_*	*R^2^*	*K_f_*	*n*	*R^2^*
Cr(VI)	198.94	0.015	0.99	29.55	3.84	0.82
Metal ion	TemKin model			Dubin Radushkevich model		
	*K_t_*	*A*	*R* ^2^	*β*	*R* ^2^	
Cr(VI)	0.37	30.9	0.91	2.29	0.96	

**Table 8:** Thermodynamic parameters of MCA for Cr(VI) adsorption.

Temperature (K)	Thermodynamic Parameters		
	∆G (kJ·mol^−1^)	∆H (kJ·mol^−1^)	∆S (J·mol^−1^·K^−1^)
298	2.802	7.324	15.172
303	2.727		
308	2.651		

## Data Availability

The original contributions presented in this study are included in the article. Further inquiries can be directed to the corresponding author.

## Abbreviations

Waste amidoxime chelate resin: WAR; modified material: MDM; pyrolysis activated carbon product: ACP; magnetic composite adsorbent: MCA.
